# Appendicitis—Its Surgical Treatment

**Published:** 1896-12

**Authors:** Howard Crutcher


### APPENDICITIS—ITS SURGICAL TREATMENT.

[A paper read by Howard Crutcher, M. D., before the Homœopathic Medical Society of Chicago, March 12th, 1896.]

MR. PRESIDENT AND FELLOW-MEMBERS: When invited by our President to prepare a paper for this evening upon the surgical treatment of appendicitis, I assured him, and now repeat that no attempt will be made to deal with so vast a subject in an exhaustive manner. That appendicitis is a surgical condition, there is no longer any respectable doubt. By this I do not mean that every case demands an operation, for it is true beyond dispute that an overwhelming majority of cases recover from the primary attack without surgical treatment, and would probably recover without any treatment whatever. But this is not the question at issue; the real question is "Would early surgery in all cases give better results than we now have?" According to the *American Text-book of Surgery* (Philadelphia: W. B. Saunders, 1895), primary appendicitis is fatal only once in eight times. In other words, in a thousand cases there will be only one hundred and twenty-five deaths. But most people will be inclined to agree with the surgeon that this is a tremendous death-rate. And what is the condition of the patient who has recovered from a primary attack of this malady? In order to decide this question I appealed to those who certainly ought to know—namely, the medical directors of ten leading American life insurance companies. The question was put to each, "Where an applicant for insurance presents a clear history of an attack of appendicitis, and has not submitted to operative relief, do you consider the risk as acceptable?" The replies were embodied in a paper which I presented to the Illinois Homœopathic Medical Association in May, 1895. There was no difference of opinion. Not one of the companies interviewed would accept a risk of the kind without a lapse of considerable time—an average of two years, if I am not mistaken. Life insurance companies are not in the business of rejecting acceptable risks; why do they unanimously avoid the man who some of the doctors think has "recovered" from appendicitis? Because they know from a costly experience that a reformed appendix, like a reformed gambler and a regenerated drunkard, cannot be depended upon. The tendency is toward relapse, and subsequent attacks are increasingly dangerous. So it is hardly fair to say that the mortality of appendicitis is only one in eight; the relapsing cases would swell the death-rate to far above the primary mortality of twelve per cent. The only genuine recovery is the case where the appendix has either been removed or has undergone obliterative changes. The latter cannot, of course, be determined with any degree of certainty by ordinary methods of examination.

Many medical men seem to think it an evidence of superior wisdom to appear indifferent in the presence of appendicitis; to doubt the diagnosis of the disease, to make light of its dangers, and to dwell upon their own astonishing success in dealing with it by internal medication. Six or eight successful cases, with no death, is a reassuring record, and most men are sufficiently human to be guided very largely by such excellent results.

But the next case may spoil the record, and the following one may intensify the lesson and leave a death-rate that no skillful surgeon in the land cannot heavily discount. Medical men are coming to see the logic of the situation, and I do not doubt that another decade will see the acute inflammatory lesions of the right iliac region recognized universally as coming within the domain of surgery. In support of this view I have been able to see in my own circle of professional associates a very gradual but decided change in favor of early surgical interference in all cases of appendicitis. I say "early" interference, for no one, I presume, has any shadow of doubt as to the need of interference when, unfortunately, interference is often too late.

I have within three days been called in consultation with a skillful medical man, who said that he desired my advice as to whether the moment had come for operation in a case of appendicitis. He was asked what symptoms he regarded as justifying an operation. His reply was that he should operate at the first signs of pus. How often does it happen that the signs of pus are the signs of death.

Touching the question of early laparotomy, the testimony of eminent specialists in general medicine has been sought. To each of these gentlemen the question was asked: "In a case where there is strong presumptive evidence of appendicitis do you consider an exploratory laparotomy by a skillful operator preferable to delay?"

Dr. Charles Gatchell, of Chicago, says: "Even with strong presumptive symptoms, I should delay rather than make an exploratory laparotomy."

Prof. William Pepper, of Philadelphia, does not consider it possible "to reply intelligently to a general question of that kind," and says "that every case must be decided upon its own merits."

Prof. James T. Whittaker, of Cincinnati, "cannot answer the question categorically," but says that "an exploratory laparotomy by a skillful surgeon is usually a safe procedure." Can it be said of appendicitis in any stage that it is "usually safe"?

Prof. George Royal, of the University of Iowa, says: "No; I should not advise it unless I felt sure that pus was present or forming."

Dr. L. C. McElwee, Professor of Materia Medica in the Homœopathic Medical College of Missouri, says: "I should answer 'Yes,' for I have just lost a case that might have recovered had that very thing" (which Prof. Whittaker says is usually a safe procedure) "been done. The symptoms present did not seem to justify an operation any sooner than was done, yet we found that it had better have been done before. Three of us were in consultation. Nobody attaches any blame to us, nor do we think that we are in any sense blameworthy, even in the light of the proceeding; but from a nicely practical standpoint, I think yes."

Dr. Frank Kraft, the distinguished editor of the *American Homœopathist*, says, with characteristic vigor and clearness: "By all means, operate."

Dr. T. Griswold Comstock, of St. Louis, a gentleman whose name is synonymous with honest and enlightened conservatism, says that he "should decide that an exploratory laparotomy was not only preferable to delay, but proper and certainly indicated," and that he believes his opinion to be in accord with that of advanced surgeons everywhere.

Dr. A. Leight Monroe, Dean of the Southwestern Homœopathic Medical College, of Louisville, asserts that as far as he is personally concerned, he should have an exploratory incision, and should certainly urge the same course upon his patients.

Dr. E. E. Case, of Hartford, Conn., whom I regard as one of the first prescribers of this country, and for whose opinion I entertain the highest respect, says that he believes the law of similars sufficient for these cases; that he has practiced medi cine for over twenty years, during which time he has had his share of appendicitis to deal with, that he has never lost a case and never had one operated upon.

Dr. Eugene H. Porter, of New York, editor of *The North American Journal of Homœopathy*, grasps the situation with singular clearness, and expresses his belief with peculiar force.

I quote in full : " In a case where the presumptive evidence of appendicitis was very strong—where there could be but little doubt of the diagnosis—I should certainly consider an exploratory laparotomy by a skillful operator preferably to delay. If the surgeon, by boldness, at times endangers the life of the patient, the physician, on his side, by a timid conservatism, does at times equal harm."

Dr. H. P. Loomis, of New York, would delay advising an operation, especially in the first attack, until very sure of the diagnosis.

From these expressions, emanating from gentlemen of deservedly high reputation as practitioners, it will be seen how widely personal experience and individual surroundings will influence the views of medical men. It will be observed, however, that a majority of those whose views are given are in favor of what appears to me to be the only conservative course, namely, a timely exploration. By "timely" exploration I mean an immediate operation—the next hour, the next minute, if possible—not the next day. Hours count in these cases. Many a case which might have been saved at seven o'clock in the evening has been lost at nine the next morning.

I should like to ask medical men why it is, when it is admitted by the ablest experts that no rule is known whereby the so-called harmless catarrhal appendix can be distinguished from a tube gangrenous from end to end, that a course is advised which consigns one in eight to the grave, when the immediate operation has a death-rate of less than one per cent.? That a case of appendicitis has recovered is nothing; the chances are that it will return. The man who carries an infected appendix in his abdomen has a graveyard sentence hanging over him. Surely this is no triumph for science. Medical men, too, are very anxious that the diagnosis shall be *certain* before any operation is advised; they demand more than the most skillful surgeon is able to give. Surgeons weigh the facts, balance the risks involved and choose the least dangerous path. That they sometimes have to go through blood in a righteous cause is no argument against either the blood or the surgery. The results obtained by surgery are such as ought to command the highest confidence of medical men. Mr. Frederick Treves says in his work (*Manual of Operative Surgery*, Philadelphia, Lea Bros. & Co., 1892) that he has had no death from appendectomy. I have not his record for the time intervening, but I notice a recent report (*Lancet*, January 4th, 1896) of ten consecutive cases without a death.

Prof. A. C. Bernays, the celebrated St. Louis surgeon, has done 166 appendectomies, in cases ranging from one day to six and one-half days in duration, with eleven deaths, a mortality of 6.6 per cent. It is entirely unnecessary to say anything as to the character of the cases which died. Notwithstanding the fact that many of his cases were perforating cases, his results are just twice as good as those obtained by medical means. As to what statistics a medical man could have shown with many of the cases saved by Bernays, may be left to the imagination. He has done a large number of operations for relapsing appendicitis, in the period between attacks, without a single death.

Prof. John B. Murphy has operated in appendicitis 293 times. In his first 207 cases he had a death-rate of nearly ten per cent. His later cases show a steady decline in deaths, solely because of earlier operation. It must be remembered that Murphy was a pioneer in appendectomy, and did many of his operations in days when a medical man had no use for a surgeon, as long as the patient was warm above the knees.

One of the most conspicuous records to be found anywhere is that of Prof. John B. Deaver, of Philadelphia, whose 200 cases, with only two deaths, and whose 140 cases without a death, are an effectual reply to the sneering imputation that surgeons are too radical, and that there is greater safety in medicine. The thing that kills is infection, and the infection develops while the attendant is waiting for—what?

I am in receipt of a recent letter from Dr. Robert T. Morris, of world-wide fame in appendectomy, which is so clear-cut and convincing that I give it in full:

"49 W. 39TH STREET, NEW YORK, March 4th, 1896.

"DEAR DR. CRUTCHER:—I have never known a death from surgery in appendicitis. Whatever deaths I have seen have occurred from septic infection, which had been allowed to get beyond the resources of the surgeon before operation was performed. In New York, to-day, the only patients who die from appendicitis can be classified as follows:

"1st. Those who neglect to call a physician.

"2d. Those who call a physician who has been too busy to inform himself upon the subject of appendicitis.

"3d. Those who call a physician who has some theory of his own on the subject of appendicitis, and who is not guided by the accurate, classified knowledge of the subject, which is to be obtained in the text-books and monographs afforded by any important medical library. Whenever a person dies of appendicitis in any of our larger cities to-day, it means that some one was to blame, and it is a case for 'asking questions.' We know that the patient would not have died if he had been given the advantages which he deserved as a citizen of a civilized community.

"Yours very truly,

"ROBERT T. MORRIS."

Prof. P. S. Connor, of Cincinnati, has had no deaths in non-infected cases.

Prof. Bayard Holmes, of Chicago, has no deaths to record from the early operation.

There is a wide difference in the classification of the various operators, but I classify my cases as extreme cases, doubtful cases, and early cases. My death-rate in the extreme cases has been 75 per cent., in the doubtful cases 25 per cent., and in the early cases no death at all has occurred. Perhaps the best exponent of modern medical opinion is the *American Year Book of Medicine and Surgery* (Philadelphia, W. B. Saunders, 1896), from which it will be seen that the drift of medical belief is toward the early operation in appendicitis. In the light of the facts it could not be otherwise. A patient divided against himself in the right illiac region—cannot stand. Appendicitis does not kill, nor the surgery, nor the shock, nor the anæsthetic; but pus, adhesions, and sepsis do. Medical experience has proven that these cases often spend weeks in bed, exposed to great danger, with chances of one death in eight. Murphy, McBurney, Morris, Shrady, Senn, Deaver, Lamphere, Bernays, Treves, Fowler, Wyeth, and a score of operators not so famous, have demonstrated by a vast experience that the surgery of appendicitis is, comparatively, not dangerous. It is our plain duty to accept the situation as backed by overwhelming facts, and remove the appendix before it removes the patient.

### Discussion.

Dr. Chas. Adams—I think that but slight exception can be taken with the ground stated by Dr. Crutcher. In all cases where, while the patient seems to be doing well there suddenly come the chill and other signs of perforation, the abdomen should be opened at once. I even think it would be better to operate with what may be at hand, rather than waiting to send for an elaborate outfit. Then we have cases of acute appendicitis, where the pulse is persistently high for more than thirty-six hours; these should be operated upon at once. I mean a pulse which runs above 120. In these cases you may wait an hour or two, but operate quickly. Then we have other cases—mild cases, cases which have a tendency to get well, sub-acute cases—and here we may delay the operation until the acute attack is over. The tendency of the day is to operate in every case; I except the mild cases. There are many people in this city who have had no interference with health, no lesion discoverable, after a mild attack of appendicitis; these cases I let alone. But when an attack is severe, when it leaves disturbed digestion or occasional spells of tenderness in the right iliac region—whether it lays them up or not—I should operate, because the next attack may be perforative or infectious. We sometimes unwisely postpone an operation because there is no tumor presenting, or because the patient seems too weak to stand the operation; and this plan frequently costs a life. When we have a case going on for some time, with trouble in the right iliac region, pulse and temperature not much out of the way, suddenly showing a chill, followed by rise of pulse and temperature, it means perforation; there may be no tumor presenting, but if we wait for the tumor we lose the patient. If the appendix be free it means general septic peritonitis, from which they sometimes, but rarely, recover. If on the other hand, the appendix be walled off by adhesions, it is still wise to operate, because you thus get rid of pus. Why not operate in every case? Because it is wiser to operate in the interval between attacks, if you can wait for it, and work in tissue which is not inflamed. I look upon the operation between attacks as a perfectly safe procedure, and have never had such a case give me the slightest uneasiness following the operation. I have lost no cases where I operated for a local abscess. I have operated upon and lost "late" cases where there was septic infection from the beginning. The operation during a slight attack is safe if done according to modern surgical principles. The practitioner is safe who removes every appendix which is damaged by inflammation.

Dr. G. F. Shears—This is a question which seems to offer the chance for a wide difference of opinions, judging from what I read and hear at different medical meetings. The therapeutist states his case very strongly; many have said that they have treated appendicitis for twenty years without a death, and cannot understand why the surgeon should advise such an early operation. I have tried to consider the matter from both standpoints, and have come to the conclusion that if I am called to see a case of appendicitis on the first day, and the symptoms are severe, I advise operation; and where such advice has been accepted and followed, the results have been very satisfactory. Where called on the third or fourth day, unless there were symptoms of perforation, I have not advised operation. The experience is that operations at such period are more dangerous. Not because of the danger of the operation in itself, but because the tissues are inflamed, the shock greater, and the general condition more unfavorable. If the patient recovers from a severe attack, I advise operation for the removal of the appendix as soon as the inflammation disappears, for I have never lost a patient from an operation during the interval, and consider it perfectly safe. There is a constant tendency to recurrence following a severe attack. If the attack is light I do not advise operation, as many of such cases get perfectly well and show no recurrence. The cases must be individualized. I have little or no confidence in the physician who says that he never saw a fatal case of appendicitis in twenty years of practice. It is like the man who has never seen a lacerated cervix or perinæum in twenty years of practice—apt to give rise to the suspicion that either he would not recognize the condition when he met it, or else has had a very limited practice. Even in such books as Arndt's *System of Medicine* (in which Dr. Cowperthwaite wrote the article on Peritonitis) it is stated that many of those cases came from appendicitis. Before the present knowledge of the organ, appendicitis was regarded as a dangerous and fatal disease; but lately some practitioners seem inclined to hedge on this statement. The disease from the start is surgical; I mean it should be considered by the surgeon. If obliged to operate on every case, I should prefer to have the chance of operating on the first day. After the third or fourth day without perforation, the patient stands a better chance without operation.

Dr. H. R. Chislett—I do not know that I can add very much to what has been said. I think it cannot be too strongly emphasized that when you see the first sign of pus there is necessity for immediate operation whether a tumor presents or not. But if you are called to a case where perforation had already taken place, and you have a tumor presenting, and if you open that pus cavity before it has had time to wall itself off by firm adhesions, you have to deal with free pus in the cavity, and no amount of bloody sponging or washing will suffice to make the intestines clean. In such cases I stand with Dr. Shears, and wait until firm adhesion have formed. After a few days the operation may be done the same as in a case of ordinary abscess, and with as little danger. There is no doubt of the advisability of the early operation in severe cases. But in mild cases, while Dr. Crutcher says than an operation done on the first day is practically free from danger, having a mortality of only one per cent., I doubt if any of the others present has had such good results from operations done during an acute attack. As Dr. Adams had said, it is not the operation which kills, but septic peritonitis or embolic infection of the liver. If the medical men can tide the patient over the acute attack the patient has a better chance from an operation during the interval. I believe no surgeon can operate during acute attacks, even of the catarrahal form, and have as low a mortality rate as one per cent.

Dr. A. G. Beebo—I do not know that I can add anything to what has been said. In fact, after Dr. Shears' remark I feel almost afraid to say anything, for I am bound to confess that in my own practice I have not seen a case of appendicitis which required operation since I operated on one back in the 70's, when we called it by other names. Ever since I have been waiting in vain for another operative case in my own practice. I have had cases where I thought there was inflammation there, but not sufficient to require operation, and none of these cases have relapsed and afterward demanded operation. I have been called in consultation to see many cases of supposed appendicitis; sometimes it was appendicitis and sometimes it was not. Sometimes I advised operation and sometimes not. I do not see how I could establish a rule for date of operation by the pulse, temperature, or duration of the disease. I should have to be guided by the symptoms in each individual case. If I was satisfied that there was pus forming, or inflammation which would result in it, I should advise operation. I have had some of the common experience in operating on the late cases. I remember losing one case where there was general septic infection, and, though the chance is small, I don't know that I should not operate under similar conditions if called to-morrow. I cannot escape the suspicion that there have been a great many bellies cut open for removal of the appendix where there was no appendicitis, or no condition demanding operative interference. If such is not the case, it seems that even with the moderate opportunities at my command, I should have seen more of the other sort. My sympathies are somewhat with the medical men. Maybe I have the reputation of being old-fogyish, but I want to see good and sufficient cause for going into an abdomen before I open it. I know it is easy to tell the friends of the patient that the operation was absolutely necessary after getting into the abdomen and finding it was not, but that is a sort of surgery for which I do not hanker. I should rather have a clear conscience.

Dr. J. J. Thompson—I do not like to have to disagree with the opinions of so many eminent surgeons, but this is a question which has two sides. Some of us are a little too free with the knife in these cases. Even granting that Dr. Crutcher is entirely right, and that Dr. Adams and the rest of these careful and experienced surgeons are correct, and lose only such a small proportion of their cases, I still believe that there are more people killed by the operation of appendectomy than are cured by it. I know that it is held to be a very easy and safe operation, by the essayist and by many of the books; so, every time a patient has a bellyache, some recent graduate or unskilled surgeon like myself, is tempted to open the abdomen. A diseased appendix is found and removed, and death results. Now if those cases would go to Adams or Crutcher, it would be all right, but the fact is that the average surgeon does not get any such results. Some months ago a patient was sent to me from a suburban town, to have his appendix removed; I performed the operation, finding the intestine gangrenous and the patient died. I blamed his physician for not having had the operation done sooner. He said, "What are we country doctors to do? Just recently a young Chicago physician was taken with supposed appendicitis, operated upon by one of our surgeons, and died almost immediately." A few weeks ago I was invited to witness an appendectomy; I went; for, like Dr. Beebe, I am watching these cases with both eyes, and operate in every case where my conscience will allow. This surgeon cut into the right iliac region and hunted for an appendix for thirty minutes, but could not find any sign of it. I understand that the essayist on one occasion failed to find the organ. (Dr. Crutcher, "That's so"). Now here are two cases where no appendix could be found. About two weeks ago, I received a telegram from a Wisconsin town, saying "Acute appendicitis; come at once." In this case we had every evidence of appendicitis, and the case seemed hopeless. I cut down on a mass of pus, evacuated about a pint, douched carefully with Peroxide, and then found a perfectly normal appendix and cæcum. The patient, a young man, while coming home from school, fell so the corner of his dinner bucket struck him in the side, causing a traumatic abscess. In many cases we have no appendicitis, or if we do, the operation could have been avoided by treatment. The oil treatment, with Belladonna and Bryonia will give better results in many cases. The surgeons say, that if taken in time, 99 per cent. can be cured by operation. I would almost say that, if taken in time, 101 per cent. can be cured by the indicated remedy. Few of them are taken in time, so surgery is sometimes imperatively necessary. I saw an operation by one of our north side surgeons, a bright, careful, conscientious man, but when the appendix was removed, and subjected to examination, I could see nothing wrong with it. There was abdominal pain and tympanitis, but I could detect no pathology in the appendix. To-night we passed resolutions condemning the action of the City Board of Health in pursuing an arbitrary course in regard to the treatment of certain diseases. It seems to me, that, as homœopaths, we cannot afford to indorse a paper which advises cutting down upon a pain in the region of the vermiform appendix.

J. D. Craig, M. D.—I have never operated for appendicitis. When I had my first case it was so long ago that no one thought of opening the abdominal cavity. I have had perhaps a half-dozen cases which I diagnosed as appendicitis, and have lost one. If, as is alleged, many cases, diagnosed as colic, peritonitis, etc., are really appendicitis, the success of the medical treatment is greater than claimed. I should not hesitate to operate if I deemed it necessary. My last case was in the person of my son-in-law, who had three or four acute attacks, but the temperature did not run very high. It has been over a year since the last attack, and I think he will probably have no more. Taking it altogether, those who pay careful attention to the indicated remedy have as good results as those who resort to surgery.

Dr. H. P. Skiles—I should like to ask the essayist if at the time of draining an appendicular abscess he also removes the appendix, or whether he makes a second operation for the removal of the appendix, and if so, how long does he wait? In these cases the hardest point for me is the diagnosis. Three or four years ago I had a case, apparently of appendicitis, and it ran along for some five days, hoping to get along without an operation. I called as counsel another, and, I supposed, a better man, who said it was not appendicitis at all. The result was that I was discharged inside of twenty-four hours. My successor operated and evacuated a lot of pus. Three or four weeks ago this patient had a recurrence, was operated upon a second time by the same surgeon, and died.

Dr. W. W. Stafford—I have had no bitter experience with appendicitis, excepting that connected with the late operation. The early operation has yielded the most satisfactory results, even when performed at the height of the inflammation. I call any operation "early" if performed before perforation, and the results in such cases have left nothing to be desired. I have heard of, but have never seen nor had in my practice, a death from the early operation. The death-rate from such operation is stated as one out of one hundred. You may almost reverse the figures where you have to deal with free pus and a weak or general septic infection, leading to secondary abscesses, fœcal fistulæ, rotten intestine, and death. Dr. Thompson has said that the recent graduate or the unskilled surgeon is apt to take the word of some one that appendectomy is an easy and safe operation, and to do more harm than good. Allow me to suggest that the unskilled practitioner, with his expectant treatment and crude prescription, is apt to do more harm than he would with a knife. I remember one case in particular—recurrent appendicitis. The first attack I did not see. About a year later I treated her at the hospital during the second attack, from which she recovered nicely, without evidence of abscess. About one year after this the third attack came suddenly, and was in full bloom when I was called. The house was remote from my office, and I hesitated between operating at that time, with the impossibility of closely watching the case afterward, and the medical treatment with its chance of a forced operation, possibly at night, with insufficient help, light, and unfavorable surroundings. However, I risked the case on medicine, and at the end of a slow convalescence she went to the hospital, and I removed the offender. I found that, with the exception of a quarter of an inch at the distal extremity and a narrow ring of tissue at the appendo-cæcal junction, the mucous lining, the submicosa, and almost all of the muscularis were ulcerated away, leaving only a few strands of muscular tissue and the peritoneum between the lumen of the appendix and the peritoneal cavity. I had won, but by an uncomfortably small margin. This question came up at the last summer's meeting of the International Hahnemannian Society on a discussion of a paper on "Appendicitis" by that able homœopathic surgeon, Dr. J. B. Bell, of Boston. In that discussion Dr. Bell, than whom there are few homœopaths in this country better qualified to pick the *indicated* remedy, said that if his remedy did not produce decided improvement within twenty-four or forty-eight hours he operated. I think this a safe rule to follow in all early cases where the symptoms are not very bad. Personally, I should prefer to err on the side of safety, the early operation, with its insignificant death-rate, than on the safe side of the expectant treatment, an indifferent prescription, and a mortality of twelve out of one hundred.

Dr. A. G. Beebe—As to statistics—nothing on earth is more misleading. There is no reliable way of making statistics in appendicitis. Some one with a large hospital practice gives us the results of his work, and they are published, and we accept them as of universal application. There is nothing which goes wider of the mark. We may see in some newspaper that there are 748,641 eggs eaten in Chicago in forty-eight hours; they may all be hatched out of the fertile imagination of some newspaper reporter.

Dr. Charles Adams—Many cases are reported as cured which are not cured; they are liable to repeated attacks. I know one patient who had thirteen attacks without an abscess; the fourteenth time there was an abscess, and I operated. I advised an operation on a friend of his who had an abscess with the first attack, but he objected on the ground that his friend had ten attacks. I operated, notwithstanding, and showed the pus to the friend. The latter had three more attacks, but the fourth, making his fourteenth, brought an abscess, so I operated. The patient who recovers should be watched, and if there is an upset digestion or impaired health following the recovery, he is liable to recurrence at any time, and you can never tell which attack will bring pus and perforation. I have never operated where there was nothing the matter with the appendix. In opening one of these abscesses it is not worth while hunting around for the appendix unless it presents; the danger lies in breaking up the adhesions, and the operation is usually not an appendectomy at this time. The favorable results quoted refer more particularly to the early operation, and not to those which dealt with perforation and pus.

G. F. Shears, M. D.—I want to say that as a result of my own experience I do not hunt for the appendix in a pus cavity, nor do I hunt for anything else; sometimes I do not even flush out the pus cavity, for fear of breaking down adhesions. I have worried over some of the cases I have had, and the matter of conscience has made me less conservative in these cases than I was when I started out. I have lost a few cases after advising against an operation, and the post-mortem made me feel that I had been to blame in failing to operate. It is sometimes a matter of conscience to those who operate, as well as with those who make it a rule not to operate.

Dr. Howard Crutcher, essayist—I would say for the benefit of Dr. Thompson, that I purposely avoided the mention of anything relating to the technique. When pus is found it is a mighty good rule to drain it and not risk breaking adhesions by hunting for the appendix; that may be left for a secondary operation. As for waiting three or four days, I was recently called in consultation by Dr. Tracy, whose patient had been in bed for ten days. After an examination I diagnosed appendicitis with possibly kidney complication. Dr. Tracy thought an operation advisable and I was in doubt, so we decided to take a look at it, and found the appendix carcinomatous. I do not know whether this operation would be deemed too early. The condition had extended to cæcum and colon. Patient made a good recovery, but has a hereafter. As to patients being killed by the operation, that is nonsense. I have operated in two cases where I could not account for the death of the patient, but an examination revealed infection. I have a collection of appendices, each with a history, which I should like to show to any one who thinks he can tell all about the condition of the appendix from the outside. I was called to see a man who had been initiated into a lodge. He felt a little indisposition, but not sufficient to call a doctor; when he did finally call a physician he was not deemed sick enough to require medicine. He grew worse in the night, I was called, operated, and removed an appendix seven inches long and packed with fæcal concretions; he very promptly died. A man came into the office one afternoon to pay his bill, and merely mentioned some symptoms which seemed to me suspicious; I examined him and diagnosed appendicitis. I was going to Kansas City, so operated upon him the next morning, and removed a short, thick, stubby, gangrenous appendix, filled with pus and certainly within forty-eight hours of perforation. I had a case a month ago in a man of fifty, and was afraid to operate because of bad kidneys; the symptoms subsided and I backed out. A couple of weeks later he had another spell, so we removed the appendix, which was not as bad as some I have seen, but I have yet to see an appendix removed too early. I think Dr. Shears' advice as to waiting three or four days is faulty, as it is as hard to tell any more about it on the fourth day than on the first or sixth day. I do not know of any rule to tell on which day to operate; the further off from the initial attack the more deaths you have, for perforation may come while you are waiting. I was called to see a case on the east side; I examined the patient, who had a history of violent colic for several days. Pulse, 80; patient resting easier and had gone peacefully to sleep. The patient was removed to the Chicago Homœopathic Hospital; an incision revealed the fact that the belly was brimful of pus. The patient died, yet at the start he did not look like a very sick man. A young man came to my office one afternoon to be examined for a supposed rupture; I called it appendicitis. He vomited two or three times while at the office, and I think that perforation had already taken place. He died before the next morning without an operation. I had two cases charged against me where I operated without finding a bad appendix. A woman fell off a street car and some queer abdominal symptoms resulted. I did not diagnose appendicitis, but thought I would find out; it was better than putting the case off with the possibility of having a funeral. I have known of these cases being diagnosed as lung fever, gall-stones, impaction of the colon, stone in the bladder—everything except appendicitis. The most picturesque diagnosis I ever heard of is on record in one of our suburbs, and was stated as "abdominal dyspepsia." Inasmuch as dyspepsia rarely occurs in the pelvic cavity, and very seldom manifests itself upon the skin, I think it was a case of cranial dyspepsia on the part of the doctor. Yet the man who made that diagnosis has criticised some of my operations. I had examined this case twice, and there is no doubt that it is appendicitis. These dyspepsia cases are dangerous and sometimes perforate when you least expect it. So, that while I think the rule has its exceptions, yet, as far as a rule goes, it is well to remove the appendix before the appendix removes the patient.

